# Spectrum of MRI findings of foetal alcohol syndrome disorders—what we know and what we need to know!

**DOI:** 10.1259/bjro.20210063

**Published:** 2023-03-28

**Authors:** Saad Sharif, Naeha Lakshmanan, Farhana Sharif, Stephanie Ryan

**Affiliations:** 1 SHO, Beaumont Hospital, Dublin, Ireland; 2 School of Medicine, Royal College of Surgeons, Dublin, Ireland; 3 Consultant Paediatrician, Mullingar Regional Hospital, Co.Westmeath, Ireland; 4 Consultant Radiologist, CHI Temple Street, Co. Dublin, Ireland

## Abstract

The exposure to alcohol *in utero* has been known to damage the developing foetus. Foetal alcohol spectrum disorders is an umbrella term that highlights a range of adverse effects linked to alcohol exposure *in utero*. Multiple studies have shown specific brain abnormalities, including a reduction in brain size, specifically in the deep nuclei and cerebellum, and parietal and temporal lobe white matter changes. While studies ascertained that other prenatal risk factors, such as maternal use of illicit drugs or lack of pre-natal care, and post-natal risk factors, such as physical or sexual abuse and low socioeconomic status, may be involved in the pathology of variances in foetal neurological abnormalities, prenatal alcohol exposure remained the strongest factor for effects on brain structure and function. Particularly, the number of days of alcohol consumption per week and drinking during all three trimesters of the pregnancy indicating the strongest relationship with brain abnormalities. Further studies are needed to explain pre-natal risk factors in isolation as well as in combination for neurodevelopmental outcomes. The diverse phenotypic presentations described indicate that the diagnostic criteria of foetal alcohol spectrum disorder must be refined to better represent the range of neurologic anomalies.

Exposure to alcohol *in utero* has been known to damage the developing foetus. Foetal alcohol spectrum disorders (FASDs) is an umbrella term that highlights a range of adverse effects linked to alcohol exposure *in utero*. Foetal alcohol syndrome (FAS) is a subset of FASD with a unique set of facial and neurological anomalies.^1^ The facial characteristics most commonly associated with FAS include a smooth philtrum, epicanthal folds, thin upper lip, short palpebral fissures, and micrognathia.^2^

There are multiple classifications available for diagnosing FASD. One endorsed by the American Academy of Paediatrics is the 4-digit diagnostic code by Susan Astley from the University of Washington. This code ranks the severity of growth deficiency and FAS facial features, the likelihood of CNS damage, and the risk of pre-natal alcohol exposure in order to precisely describe the outcomes of the large spectrum of phenotypic variants in FASD.^3^ As per Dr Astley, not all children with FASD exhibit structural or functional brain dysfunction. Thus, the term FASD is now being used to emphasize the spectrum of deficits that can be seen with this condition. The brain abnormalities can range from subtle changes at cellular level to gross structural abnormalities and behavioural and cognitive delay can range from mild to severe.^1^

Lange et al in a systematic review published in JAMA highlighted that FASD was prevalent regardless of lower socioeconomic status, but recognised Ireland as the country with the third highest rate of FASD of the 187 countries included at 47.5 per 1000 children in the general population. This is significantly higher than the global prevalence of 7.7 per 1000 children and the European prevalence of 19.8 per 1000 children.^4^ Studies found that the majority of females consume alcohol during the periconceptual period. The American Academy of Pediatrics emphasised that there is no safe amount of alcohol consumption throughout the entire gestational period.^5^ In 2019, the Health Service Executive in Ireland (HSE) issued guidelines which clearly stated that there is no safe amount of alcohol or time to consume alcohol during pregnancy. Yet, the incidence of *in utero* complications of alcohol consumption has remained high in Ireland.^6^

A national survey of paediatricians suggested an even more serious concern. Clinicians may not contemplate the diagnosis of FASD if the child is non-dysmorphic, as a significant number of clinicians are not aware of the non-dysmorphic phenotype of FASD.^7^ Given that the diagnosis of FASD without classical features can be quite difficult, it is imperative that mild to moderate phenotypes of FASD as well as the functional and behavioural deficits of lesser severity in FASD are well understood and can be recognised by clinicians.

Exposure to alcohol *in utero* can have significant outcomes for the foetus; however, not all cells within the embryo are equally affected.^8^ Animal studies have shown that the most affected cells are the cranial neural crest cells. These affect the developing facial features of the foetus.^9^ Alcohol also affects the structural as well as functional components of the brain.

Multiple studies have shown particular brain abnormalities, including a reduction in brain size and parietal and temporal lobe white matter changes. One study in particular, showed that those with facial characteristics of FAS had a greater reduction in frontal lobe size. This study also showed that an increased exposure to alcohol *in utero* resulted in a larger reduction in size of the noted brain regions, further confirming the causational relationship between alcohol consumption during pregnancy and structural brain abnormalities.^1^ A more recent study by Astley et al showed that in fact pre-natal alcohol exposure was the most prominent factor in abnormalities of brain size, brain lobes, and brain function, including executive-functioning, intellect, and behaviour. This study acknowledged that females who consume alcohol during pregnancy often have other comorbidities and considered pre-natal risk factors. However, even when correcting for maternal illicit drug use, and post-natal risk factors, such as physical or sexual abuse, pre-natal alcohol exposure remained the strongest risk factor for abnormalities in brain structure and function, with number of days alcohol was consumed per week and drinking during all three trimesters of the pregnancy indicating the strongest relationship with brain abnormalities.^10^

A retrospective study published in 2017 analysed the autopsies of foetuses, infants, children and some adults with pre-natal alcohol exposure or a diagnosis of FASD concluding that pre-natal exposure to alcohol increases the risk of spontaneous abortion, stillbirth, as well as infant and child mortality. The study showed that while organ anomalies of various degrees were noted in the different age groups, diagnostic facial anomalies for FASD were only mentioned in a minority of cases. Microencephaly, although the most common structural abnormality of the brain, was also found in only a minority of cases. The study noted that the timing at which the exposure to alcohol occurred *in utero* correlated with particular neural malformations based on the particular embryological process occurring at that time. Uteroplacental insufficiency secondary to alcohol exposure, causing hypoxic-ischemic lesions, also factored into a number of deaths.^11^

While previously post-mortem studies were required to understand the structural brain abnormalities of FASD, imaging tools are now being used for the same purpose.^2^ Thus, being aware of the radiological implications of imaging in the diagnosis of FASD is important.

Susan Astley’s study, looking at MRI outcomes of children with a range of phenotypes under the broad category of FASD, showed that functional impairment correlated with structural abnormality seen on MRI scans. Of significance, the study indicated that while infants may have neither the facial features of FAS nor microencephaly, they may still have structural brain abnormalities.^1^

Structural MRIs are able to show specific changes in volume and shape over time in children affected by FASD. While most studies show an overall reduction in grey and white matter throughout all lobes as well as a global reduction in cortical thickness, a few studies have shown increased cortical thickness and grey matter density in particular areas, including the parietal regions. The studies also showed that the rate of development differed in those with FASD when compared to their peers, with some showing a decrease in cortical volume and others showing a slower rate of thinning of the cortex over time.^2^

A 2001 study used structural MRIs to analyse the differences in brain structure amongst children with, pre-natal exposure to alcohol (PEA) with and without the FAS phenotype, and with those of controls. The groups differed significantly in cranial volumes and in cerebral and cerebellar vault volumes as well as the cerebral grey and white matter volumes, while there were no difference in regards to CSF volume. Furthermore, differences were significant between the FAS group and control group, but not between those with PEA and the control group. The particular patterns of structural malformations in the brain, found through MRI, indicate asymmetric effects of alcohol consumption on specific tissues and their development.^12^

Treit et al compared findings on routine clinical MRI of the brain in children with PEA with those of a control group. While the incidence of non-clinically significant and of clinically significant incidental findings was higher in the PEA group than in the controls, routine clinical MRI did not reveal a consistent pattern of brain abnormalities that can be used diagnostically for FASD.^13^

Diffusion tensor imaging is an MRI technique that analyses microscopic structural changes in tissues. Diffusion tensor imaging studies of FAS patients were able to detect microstructural abnormalities in milder cases without typical phenotypic characteristics. Magnetic resonance spectroscopy determines the level of neurometabolites, an indicator of the structural stability of cell membranes, and functional MRI determines the neuroactivation in particular regions of the brain. Thus far, neither have been widely used for FASD cases. Preliminary data, however, is promising in that these studies would be able to identify patterns in metabolic changes in magnetic resonance spectroscopy studies and patterns in network activity in functional MRI studies.^2^ Imaging can be a vital tool for diagnosing FASD, but in order for it to be an effective tool, establishment of what is normal in the imaging of brain development is crucial.^1^ The 2007 study by Waber details the longitudinal development of brain structures from birth to young adulthood in healthy children, creating a crucial baseline.^14^

In conclusion, children who are exposed to alcohol *in utero* can display a wide range of phenotypic effects, functional outcomes and abnormalities in brain structure.^13^ Imaging modalities should be better utilised to diagnose FAS and track the phenotypic changes with alcohol exposure *in utero*. However, the lack of clear direct teratogenic effects of ethanol *in utero* also indicate the importance of considering contributing risk factors, such as uterovascular insults and complications of prematurity.^11^ Further studies are needed which can explain these risk factors in isolation as well as in combination for neurodevelopmental outcomes. These studies make it clear that the diagnostic criteria of FASD must be refined to incorporate the diverse phenotypic presentation of the anomalies found.
